# Inhibition of PKCγ phosphorylation protects against cerebral ischemia-reperfusion injury

**DOI:** 10.1016/j.redox.2026.104221

**Published:** 2026-05-27

**Authors:** Chenchen Li, Jinlun Chen, Xiangbin Ouyang, Ruijia Duan, Yijin Kuang, Yaohui He, Jieqiong Tan, Liuwang Zeng

**Affiliations:** a Department of Neurology, The Second Xiangya Hospital, Central South University, Changsha, Hunan, China; b Clinical Medical Research Center for Stroke Prevention and Treatment of Hunan Province, Department of Neurology, The Second Xiangya Hospital, Central South University, Changsha, Hunan, China; c Xiangya Nursing School, Central South University, Changsha, Hunan, China; d MOE Key Lab of Rare Pediatric Diseases, Hengyang Medical School, University of South China, Hengyang, Hunan, China; e Center for Medical Genetics and Hunan Key Laboratory of Medical Genetics, School of Life Science, Central South University, Changsha, Hunan, China; f Hunan Key Laboratory of Animal Models for Human Diseases, School of Life Science, Central South University, Changsha, Hunan, China; g MOE Key Lab of Rare Pediatric Diseases, School of Life Sciences, Central South University, Changsha, Hunan, China; h NHC Key Laboratory of Birth Defect for Research and Prevention, Hunan Provincial Maternal and Child Health Care Hospital, Changsha, Hunan, China

**Keywords:** PKCγ, Cerebral ischemia-reperfusion, Mitochondria, Phosphorylation, Nrf2

## Abstract

Cerebral ischemia-reperfusion (I/R) injury is a major cause of stroke-related mortality and disability, primarily driven by mitochondrial dysfunction, oxidative stress, and apoptosis. In this study, we identified phosphorylation of PKCγ at the T655 site following cerebral I/R injury using mass spectrometry. Notably, we observed that approximately 5% of total PKCγ translocates to mitochondria following I/R injury, suggesting a direct role in modulating mitochondrial function. We further investigated the functional role of PKCγ both *in vitro* and *in vivo*. Our results demonstrate that the regulatory effects of PKCγ on Nrf2 and mitochondrial function depend on its kinase activity, as evidenced by the lack of effect of the kinase-dead G360S mutant. The phospho-mimetic T655D mutant suppressed Nrf2 nuclear translocation, promoted mitochondrial ROS production, fragmentation, and neuronal apoptosis, whereas the dephospho-mimetic T655A mutant exerted the opposite effects. Nuclear/cytoplasmic fractionation and immunofluorescence analyses further confirmed that PKCγ regulates Nrf2 nuclear translocation in both HeLa cells and primary neurons. Knockdown of PKCγ *via* shRNA *in vitro* and AAV9-mediated delivery in mice alleviated mitochondrial dysfunction, reduced infarct volume, and improved neurological outcomes. Behavioral assessments further confirmed the neuroprotective effect of PKCγ knockdown *in vivo*. Collectively, our findings identify T655 phosphorylation as a key mechanism by which PKCγ regulates mitochondrial dysfunction and oxidative stress during cerebral I/R injury, suggesting that targeting this pathway may represent a promising therapeutic strategy for ischemic stroke.

## Highlights

• PKCγ is phosphorylated at threonine 655 (T655) following cerebral ischemia-reperfusion injury.
• T655 phosphorylation of PKCγ modulates Nrf2 activity and mitochondrial function during ischemia-reperfusion.
• The phospho-mimetic mutant PKCγ T655D enhances mitochondrial ROS generation, fragmentation, and neuronal apoptosis after ischemia-reperfusion.
• PKCγ knockdown mitigates mitochondrial dysfunction, reduces infarct volume, and improves neurological outcomes *in vivo* following ischemia-reperfusion injury.

## Graphical abstract

Diagram of the role of PKCγ in I/R Injury.

**Figure:**
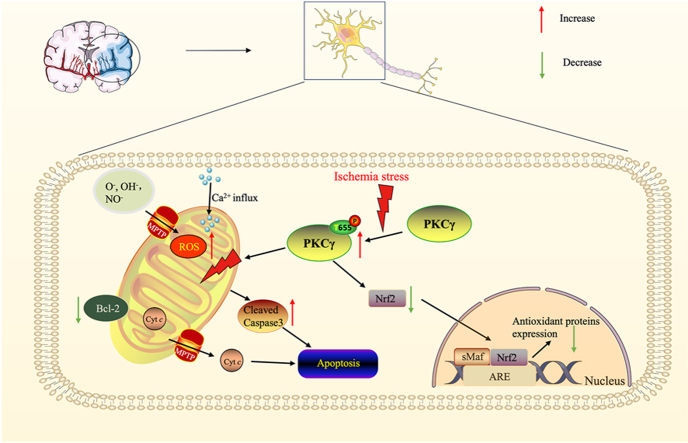

## Abbreviations:

I/R: Ischemia-reperfusion
PKC: Protein kinase C
OGDR: Oxygen-glucose deprivation/reoxygenation
Nrf2: Nuclear factor-erythroid 2-related factor 2
Drp1: Dynamin-related protein 1
MCAO: Middle cerebral artery occlusion
CCA: Common carotid artery
ICA: Internal carotid artery
ECA: External carotid artery
qRT-PCR: Quantitative real-time PCR
AAV: Adeno-associated virus
DAG: Diacylglycerol
sMaf: Small Maf
ARE: Antioxidant response element
Hsp90: Heat-shock protein 90 (Hsp90)

## 1 Introduction

Ischemic stroke is the most common type of stroke and has become one of the leading causes of death and disability worldwide [1]. Currently, the most effective treatment methods to improve the outcome of acute ischemic stroke are thrombolytic therapy and intravascular interventional therapy within the time window to restore vascular recanalization [2]. However, reperfusion injury following recanalization causes neuronal dysfunction, which seriously affects functional recovery in patients [3]. Neuroprotective therapy is an important means to reduce I/R injury [4]. Up to now, the therapeutic effect of cerebral I/R has not been satisfactory due to the lack of effective intervention targets and neuroprotective drugs. Therefore, it is particularly important and urgent to find effective targets and neuroprotective agents to reduce reperfusion injury after vascular recanalization [5,6].

Neuroprotective strategies for I/R injury include targeting excitotoxicity, oxidative stress, neuroinflammation, and apoptosis, with mitochondrial targeting being a crucial therapeutic approach [[7], [8], [9], [10]]. Mitochondria are the central organelles for cellular energy metabolism, and the process of maintaining their internal homeostasis is highly complex, involving processes such as fission and fusion [11,12]. Mitochondria are highly sensitive to ischemic damage, and inhibiting mitochondrial fission can impede the release of cytochrome *c*, delaying the process of apoptosis [13]. Mitochondrial oxidative stress leads to upregulation of the mitochondrial fission protein dynamin-related protein 1 (Drp1), causing imbalance in mitochondrial dynamics, leading to mitochondrial dysfunction, disintegration, and ultimately neuronal death [14]. Antioxidants such as MitoQ or vitamin E can reduce the expression of Drp1, decreasing mitochondrial fragmentation [15,16]. Therefore, mitochondrial proteins play a crucial role in the pathological process of cerebral I/R.

Protein phosphorylation is a critical post-translational modification involved in various biological processes, such as transcription regulation, apoptosis regulation, DNA damage repair, and signal transduction [17]. Phosphorylation modification of mitochondrial proteins is an early event in the occurrence of cerebral I/R injury [18]. Damaged mitochondria in cerebral I/R are cleared through mitochondrial autophagy, and BNIP3L, located on the outer membrane of mitochondria, serves as a receptor for mitochondrial autophagy [19]. BNIP3L inhibits and alleviates cerebral I/R injury, with phosphorylation modification at BNIP3L-Ser81 being a key factor in its involvement in mitochondrial autophagy and exerting a neuroprotective effect [20]. Therefore, phosphorylation modification of mitochondrial proteins may play a role in the pathological process of I/R injury. Despite the presence of over 1000 mitochondrial proteins, the dynamics and roles of phosphorylation modifications during cerebral I/R remain largely unexplored [21]. Protein kinases, which are themselves regulated by phosphorylation, offer attractive targets for modulating these modifications and for therapeutic intervention. Among protein kinases, members of the protein kinase C (PKC) family have been implicated in the regulation of oxidative stress responses through modulation of Nrf2 signaling [22]. For example, PKCα and PKCδ have been reported to phosphorylate upstream regulators of Nrf2 or influence Nrf2 stability and nuclear translocation, thereby contributing to cellular antioxidant defense [23]. However, compared with other isoforms, the role of PKCγ in regulating Nrf2 signaling in the brain remains largely unclear, particularly regarding its subcellular localization-dependent functions.

In this study, we performed phosphoproteomic analysis of brain mitochondria from mice after cerebral I/R injury and identified a significant increase in PKCγ phosphorylation. In particular, phosphorylation at the T655 site was markedly upregulated following oxygen-glucose deprivation and reperfusion (OGDR) *in vitro*. Given the emerging role of PKC family members in regulating oxidative stress pathways, including Nrf2 signaling, we further investigated the functional relevance of PKCγ T655 phosphorylation. Functionally, PKCγ knockdown protected against OGDR-induced mitochondrial fragmentation and neuronal apoptosis. To further investigate the role of T655 phosphorylation, we generated kinase-dead (G360S), phospho-mimetic (T655D), and dephospho-mimetic (T655A) PKCγ mutants. Our results demonstrated that T655 phosphorylation regulates PKCγ kinase activity, thereby indirectly modulating Nrf2 signaling and mitochondrial homeostasis. Notably, *in vivo* knockdown of PKCγ using AAV delivery reduced infarct volume and improved neurological outcomes in mice. These findings reveal a critical role for PKCγ T655 phosphorylation in I/R-induced neuronal injury and highlight its potential as a therapeutic target.

## 2 Materials and methods

### 2.1 Reagents and antibodies

Antibodies for Cleaved Caspase-3 (#9664), Tom20 (#42406), Phospho-(Ser/Thr) Phe (pan P) (#9631), Flag (#8146), GFAP (#80788) and Histone H3(#4499) were purchased from Cell Signaling Technology (MA, USA), Bcl-2 (#ab182858) was purchased from Abcam (Cambridge, UK). PKCγ (#66429-1-Ig), Nrf2 (#16396-1-AP), VDAC1 (#55259-1-AP), Calnexin (#10427-2-AP), β-actin (#66009-1-Ig) and GAPDH (#60004-1-Ig) were obtained from Proteintech Group, Inc (Wuhan, China). Iba1 (#019-19741) was purchased from FUJIFILM Wako Pure Chemical Corporation (Osaka, Japan). NeuN (#MAB377) and MitoSOX™ Mitochondrial Superoxide Indicators, for live-cell imaging (#M36006 and M36008) were purchased from Thermo Fisher Scientific (MA, USA). Cycloheximide (CHX) (#S7418), the protease inhibitor cocktail (#B14001) and phosphatase inhibitor cocktail (#B15001) were purchased from Selleck Chemicals (TX, USA).

### 2.2 Cell culture

The mouse neuroblastoma N2a (N2a) cells (catalog, CCL-131) and HeLa cells (catalog, CCL-2) were purchased from American Type Culture Collection (ATCC) (VA, USA). The N2a cells were cultured in DMEM/F-12 (1:1) complete culture medium (Sigma-Aldrich, MO, USA; #C11330500BT) with 10% Fetal Bovine Serum (FBS) (Sigma-Aldrich, MO, USA; #F8318), MEM NEAA (Sigma-Aldrich, MO, USA; #11140-050), l-Glutamine 200 mM (Sigma-Aldrich, MO, USA; #25030-081), Sodium Pyruvate 100 mM (Sigma-Aldrich, MO, USA; #11360-070), penicillin (50 U/mL) and streptomycin (50 μg/mL) (Sigma-Aldrich, MO, USA; #15140-122) in a humidified incubator at 37 °C with 5% CO_2_. HeLa cells were cultured in complete DMEM medium (Sigma-Aldrich, MO, USA; #C11995500BT) containing 10% FBS.

### 2.3 Primary cortical neuron culture

Primary cortical neurons were obtained from E16 mouse embryos. After anesthetizing and euthanizing the pregnant mouse, embryos were extracted, and brain tissues were isolated in HBSS (Thermo Fisher Scientific, MA, USA; #14175095) with careful removal of blood vessels. The cortical tissue was finely minced using scissors and digested with Papain (Worthington Biochemical Corporation, NJ, USA; #LS003126) for approximately 12 min. Digestion was then neutralized with MEM (Thermo Fisher Scientific, MA, USA; #11095080) containing 10% FBS. The cell suspension was filtered through a 40 μm cell strainer (BD Falcon, CA, USA; #352340), centrifuged, and the supernatant was discarded. The pellet was resuspended in MEM containing 10% FBS. After cell counting, neurons were plated onto Poly-d-Lysine (Sigma-Aldrich, MO, USA; #A-003-E)-coated 24-well plates and incubated at 37 °C in a humidified incubator with 5% CO_2_. After 4 h, the medium was replaced with Neurobasal medium (Thermo Fisher Scientific, MA, USA; #21103) supplemented with 1% GlutaMAX (Thermo Fisher Scientific, MA, USA; #10565018) and 2% B-27 Serum-Free Supplement (Thermo Fisher Scientific, MA, USA; #17504-044). Experiments were conducted on days 10-12.

### 2.4 Mitochondria isolation from mouse brain tissue

Mitochondria were isolated from mouse brain tissue using a modified Percoll (Sigma-Aldrich, MO, USA; #P1644) density gradient centrifugation method as previously described by Sims et al. [24]. Briefly, following euthanasia by decapitation, brain tissues were rapidly collected, homogenized in ice-cold isolation buffer, and subjected to differential centrifugation. The crude mitochondrial pellet was resuspended and purified using a discontinuous Percoll gradient, yielding a fraction enriched in non-synaptosomal mitochondria. All procedures were performed at 4 °C to preserve mitochondrial integrity.

### 2.5 OGDR model

The cells were treated with OGD through replacing the culture medium with DMEM, no glucose (Thermo Fisher Scientific, MA, USA; #11966025). Subsequently, the cells were immediately transferred to a hypoxic incubator chamber (Heal Force, Shanghai, China; #HF100) with a gas mixture of 5% CO_2_ and 95% N_2_ at 37 °C for 6 h. Then, the cells were replaced with DMEM/F-12 (1:1) complete culture medium and continued to be cultured under normal conditions for reoxygenation of 24 h.

### 2.6 Immunoprecipitation (IP)

For the IP of exogenously expressed PKCγ WT/T655A Flag, 1 × 10^6^ N2a cells were transfected with either pcDNA3.1 or PKCγ WT/T655A-Flag plasmids. After 24 h of transfection, the cells were subjected to OGDR treatment and then lysed on ice for 5 min using 500 μL of Western and IP cell lysis buffer (Beyotime Biotechnology, Shanghai, China; #P0013). The lysates were further incubated with gentle rotation at 4 °C for 1 h to ensure complete lysis. Subsequently, the samples were centrifuged at 13,000 ×g for 30 min at 4 °C, and the supernatants were collected as total protein samples. An appropriate volume of each lysate was mixed with an equal volume of 4× SDS sample buffer, boiled at 95 °C for 10 min, and stored at −80 °C for later use. For the IP procedure, Anti-FLAG® M2 Magnetic Beads (Sigma-Aldrich, MO, USA; #M8823) were pre-washed twice with Western and IP lysis buffer. The washed beads were then incubated with the prepared protein lysates overnight at 4 °C with gentle rotation. On the following day, the beads were washed several times with the same lysis buffer to remove non-specific proteins. The bound proteins were eluted by adding an appropriate volume of 2× SDS sample buffer, followed by heating at 95 °C for 10 min. The eluted proteins, along with total protein lysates, were subjected to western blotting analysis.

### **2.7** *Cell viability analysis*

To test cell viability, cells were seeded in 96-well plates (5000 cells/well) according to cell growth rate and size. After treatment, the medium was removed and CCK8 (Selleck Chemicals, TX, USA, #B34302) was added to the plates according to the instructions. After incubation at 37 °C for 1 h, optical density (OD) was measured at 450 nm using a microplate reader to calculate cell viability.

### 2.8 LDH analysis

The cells were inoculated into a 96-well plate according to the growth rate and size of the cells. Following treatment, the culture medium from each well was collected, centrifuged at 400 ×g for 5 min, and 50 μL of the supernatant from each well was transferred to a new 96-well cell culture plate. An equal volume of LDH detection working solution was added, thoroughly mixed, and then the plate was incubated at room temperature in the absence of light for 30 min. Absorbance was measured at 490 nm and 680 nm using a microplate reader. LDH analysis was performed according to the instructions provided with the LDH assay kit (Thermo Fisher Scientific, MA, USA; #C20300), and the cell death rate was calculated.

### 2.9 Western blotting

Brain tissue or cells were lysed in a 2× SDS buffer containing pre-added protease inhibitor cocktail and phosphatase inhibitor cocktail. The protein content was quantified using a BCA protein assay kit (Thermo Fisher Scientific, MA, USA; #23227). Proteins were separated by SDS-PAGE gel electrophoresis at a constant voltage of 80 V for 30 min until proteins reached the separating gel, then the voltage was adjusted to 120 V for continued electrophoresis at a constant current of 290 mA for approximately 90 min to transfer proteins onto a 0.45 μm PVDF membrane (Sigma-Aldrich, MO, USA; #IPVH00010). The PVDF membrane was blocked with 5 % milk in PBST at room temperature for 1 h, then incubated overnight at 4 °C with primary antibodies diluted in 5% BSA. The following day, the PVDF membrane was incubated with HRP-conjugated secondary antibodies (Jackson ImmunoResearch Laboratories, PA, USA​) diluted in 5% milk at room temperature for 1 h. Protein bands were visualized using the ChemiDoc™ Imaging System (Bio-Rad Laboratories, CA, USA), and grayscale analysis was performed using Image J software.

### **2.10** *Mice*

C57BL/6 mice, weighing 20 ± 5 g, were obtained from the Animal Resource Center of Central South University. All animals were housed under standardized conditions (temperature: 25 ± 2 °C, humidity: 55 ± 10%), with a 12 h light/dark cycle maintained. All animal procedures were conducted in accordance with the regulations of the Animal Protection and Use Committee of the Hunan Provincial Experimental Animal Association and approved by the Animal Protection and Ethics Committee of Central South University.

### 2.11 Transient middle cerebral artery occlusion (MCAO)

Male mice weighing 20-25 g were used to establish a middle cerebral artery occlusion/reperfusion model using the intraluminal filament method. Mice were anesthetized by intraperitoneal injection of 50 mg/kg sodium pentobarbital. After anesthesia, the fur on the mouse's neck was removed, and the skin on the neck was cut using scissors. Under a stereomicroscope, the left common carotid artery (CCA), internal carotid artery (ICA), and external carotid artery (ECA) were exposed. The ECA was ligated with a surgical suture at the distal end, leaving a slipknot at the proximal end. The CCA near the heart and the ICA were clamped with arterial forceps. A small incision was made in the ECA, and a silicon-coated nylon monofilament (Beijing Cinontech Co., Ltd., Beijing, China; #1620A2) was gently inserted after releasing the slipknot at the proximal end of the ECA and the arterial forceps on the ICA. The ECA was then cut, and the filament was adjusted and gently inserted into the ICA until a slight resistance was felt. After the surgery, mice were placed on a temperature-controlled blanket to maintain a rectal temperature of approximately 37 ± 0.5 °C. After 60 min of ischemia, the filament was withdrawn to initiate reperfusion. In the sham-operated group, mice underwent the surgical procedure without filament insertion. All mice were euthanized 24 h after MCAO for analysis.

### 2.12 Lentivirus infection

N2a cells were infected with PKCγ-specific shRNAs lentiviruses and selected with puromycin 48 h later to establish stable knockdown lines. The target sequences for N2a cells were as follow: 5-CGACGAACTCTATGCCATCAA-3,5-CCAGGGCTTTACTTATGTGAA-3,5-CCACAAGTTCACCGCTCGTTT-3. The target sequences for HeLa cell were as follow: 5-TCTGTCGATTGGTGGTCCTTT-3, 5-GTGGAATGAGACCTTTGTGTT-3, 5-CAATGGTCTCTCTGATCCCTA-3). Subsequently, the cells were treated with OGD or other specified conditions.

### 2.13 Immunofluorescence staining

Immunofluorescence staining was performed on HeLa cells. Fix samples in 4% paraformaldehyde solution for 15 min, permeabilize with 0.1% Triton-X 100 in 1× PBS for 15 min, block with 5% BSA solution at room temperature for 1 h, and then incubate with the corresponding primary antibody at 4 °C overnight. The next day, wash samples in PBS and incubate with secondary antibodies conjugated with fluorescent dyes (Jackson ImmunoResearch Laboratories, PA, USA​) in 5% BSA at room temperature for 1 h. Finally, wash in 1 × PBS and incubate with DAPI (Sigma-Aldrich, St. Louis, MO, USA; #D9542) at room temperature for 5 min. Visualize immunofluorescence using Leica SP5 Confocal Microscope (Leica Microsystems, Wetzlar, Germany).

### 2.14 Detection of mitochondrial reactive oxygen species (ROS) using MitoSOX

N2a cells were incubated with 5 μM MitoSOX at 37 °C in a light-protected cell culture incubator for 30 min. After incubation, cells were washed twice with PBS to remove unbound dye. Fluorescence signals were detected using a fluorescence microscope, and fluorescence intensity changes were analyzed to assess mitochondrial oxidative stress levels.

### 2.15 RNA extraction, reverse transcription, and quantitative real-time PCR (qRT-PCR)

Following the manufacturer's instructions, total RNA was extracted from cultured cells using TRIzol reagent (Thermo Fisher Scientific, MA, USA; #15596026) and its concentration was determined by spectrophotometry. Subsequently, 1 μg of RNA was reverse transcribed into cDNA using HiScript Q RT SuperMix for qPCR (+gDNA wiper) (Vazyme Biotech Co., Ltd., Nanjing, China, #R123-01). qRT-PCR was performed using ChamQ Universal SYBR qPCR Master Mix (Vazyme Biotech Co., Ltd., Nanjing, China, #Q711-02) according to the manufacturer's protocol. Gene relative expression levels were normalized to β-actin mRNA levels, and gene expression was analyzed using the 2-ΔΔCt method. The primers used were as follows: PKCγ (human): Forward 5′-TCCTCTCCCTTCCACCTGTT-3′, Reverse 5′- TGCTTTCCGATACCCCAGAT-3′. PKCγ (mouse): Forward 5′- GAGACCTTCGTGTTCAACCTG-3′, Reverse 5′- CCCTCCTCCTGGTTCAGTAAC-3′. β-actin (human): Forward 5′-CATGTACGTTGCTATCCAGGC-3′, Reverse 5′-CTCCTTAATGTCACGCACGAT-3′. β-actin (mouse): Forward 5′-GGAGATCACAGCTCTGGCT-3′, Reverse 5′-GTCGATTGTCGTCCTGAGG-3′.

### 2.16 Plasmid construction and transfection

We purchased the pcDNA3.1 PKCγ-3 × Flag plasmid from Obio Biotechnology Co., Ltd. Primers were designed using the online tool from Agilent. We utilized a homologous recombination kit (TransGen Biotech Co., Ltd., Beijing, China; #CU201-02) to construct PKCγ T655 point mutation plasmids mimicking phosphorylation (PKCγ T655D, Threonine-to-Aspartate) and dephosphorylation (PKCγ T655A, Threonine-to-Alanine), as well as a kinase-dead mutant (PKCγ G360S, Glycine-to-Serine). The G360S mutation was reported to result in decreased or even absent catalytic activity [25]. All mutants were validated through DNA sequencing analysis (performed by Sangon Biotech Co., Ltd., Shanghai, China). Subsequently, transfection was carried out in N2a cells using Lipofectamine™ 3000 (Thermo Fisher Scientific, MA, USA; #L3000015) according to the manufacturer's instructions. Cells were subjected to OGDR treatment or other experiments after 24 h.

### 2.17 siRNA-mediated gene knockdown

For Nrf2 knockdown, N2a cells were transfected with one of three different siRNAs targeting Nrf2 (mouse) (siNrf2-1: 5′ CCGAAUUACAGUGUCUUAATT 3'/5′ UUAAGACACUGUAAUUCGGTT 3'; siNrf2-2: 5′ CGAGAAGUGUUUGACUUUATT 3'/5′ UAAAGUCAAACACUUCUCGTT 3'; siNrf2-3: 5′ GCACAAUGGAAUUCAAUGATT 3'/5′ UCAUUGAAUUCCAUUGUGCTT 3′) or a scrambled control siRNA, all purchased from GenePharma Co., Ltd. (Suzhou, China), using Lipofectamine™ 3000 (Thermo Fisher Scientific, MA, USA; #L3000015) according to the manufacturer's instructions. Knockdown efficiency was confirmed 48 h post-transfection by Western blot analysis.

### 2.18 Nuclear and cytoplasmic fractionation

Nuclear and cytoplasmic fractions were isolated using a hypotonic lysis method. Briefly, cells were trypsinized, collected by centrifugation at 800 rpm for 5 min, washed with PBS, and centrifuged at 1000 rpm for 5 min. Approximately 1 × 10^7^ cells were resuspended in 1.5 mL ice-cold 1× hypotonic buffer and incubated on ice for 15 min. Then, 75 μL of 10% NP-40 containing protease inhibitors was added, followed by vortexing for 10 s. After centrifugation at 3000 rpm for 10 min at 4 °C, the supernatant was collected as the cytoplasmic fraction, and the pellet was retained as the nuclear fraction.

### 2.19 Intracranial targeted injection of AAV

We designed adeno-associated viral vectors (AAV9) targeting PKCγ which were obtained from Obio Technology (Shanghai, China). Twenty-one days prior to MCAO surgery, stereotactic injections of adeno-associated vectors were administered to mice. The mice were anesthetized with isoflurane and placed on a stereotactic apparatus. A craniotomy was performed using a drill, and 500 nL of viral solution was injected into the left hemisphere cortex using a microinjector (Hamilton Company, NV, USA). The needle was left in place for 10 min to prevent viral reflux before being slowly withdrawn. The coordinates for the cortex were x = ± 2.00 mm, y = - 0.22 mm, z = - 1.80 mm. In this study, we used AAV9 as the viral capsid, which, upon intracerebral stereotactic delivery, predominantly transduces neurons in the cortex [26].

### 2.20 2,3,5-Triphenyltetrazolium chloride (TTC) staining

Cerebral infarction was evaluated on day 1 following MCAO using 2% TTC (Solarbio Life Sciences, Beijing, China; #G3005) staining. After euthanasia, brains were rapidly removed and placed in a mouse brain matrix. To facilitate slicing, tissues were briefly frozen at −20 °C for 15 min, then cut into 1-mm-thick coronal sections. The sections were incubated in TTC solution for 15 min, during which viable brain tissue stained red, while infarcted regions remained white. Images of stained sections were captured, and infarct size was quantified using ImageJ software. Infarct volume was calculated using the following equation [27]: Infarct volume (%) = [(contralateral hemisphere area − non-infarcted ipsilateral area)/(2 × contralateral area)] × 100%.

### 2.21 Modified neurological severity scores (mNSS) assessment

Neurological deficits were evaluated on day 1 after MCAO using the mNSS [28]. This composite scoring system includes assessments of motor function (forelimb and hindlimb flexion, head movement; 0–6 points), balance on a narrow beam (0–6 points), and reflex/abnormal movements (0–2 points), with a total score ranging from 0 to 14. Neurological impairment was classified as mild (1–4), moderate (5–9), or severe (10–14). Scoring was performed independently by three blinded investigators.

### 2.22 Rotarod test

Motor coordination and balance were assessed using the rotarod apparatus [29]. Mice were pre-trained four times per day for three consecutive days before the MCAO surgery. On the first day after MCAO surgery, the mice were placed on an accelerating rotarod (from 0 to 40 rpm over 5 min), and the latency to fall was recorded. Each mouse underwent three trials with a 15-min interval between trials, and the average fall time from the three trials was used for analysis.

### 2.23 Statistical analysis

Data are presented as the mean ± standard error of the mean (SEM) from at least three independent experiments. Statistical analysis and graphical representation of the data are conducted using GraphPad Prism 9 software. Student's t-test is used to compare the data of two groups. For comparisons among multiple groups, one-way analysis of variance (ANOVA) followed by Tukey's post hoc test is performed to identify intergroup differences. A *P* value < 0.05 was considered statistically significant.

## 3 Results

### **3.1** *PKCγ phosphorylation is increased in the early stages of cerebral I/R*

**Fig. 1:**
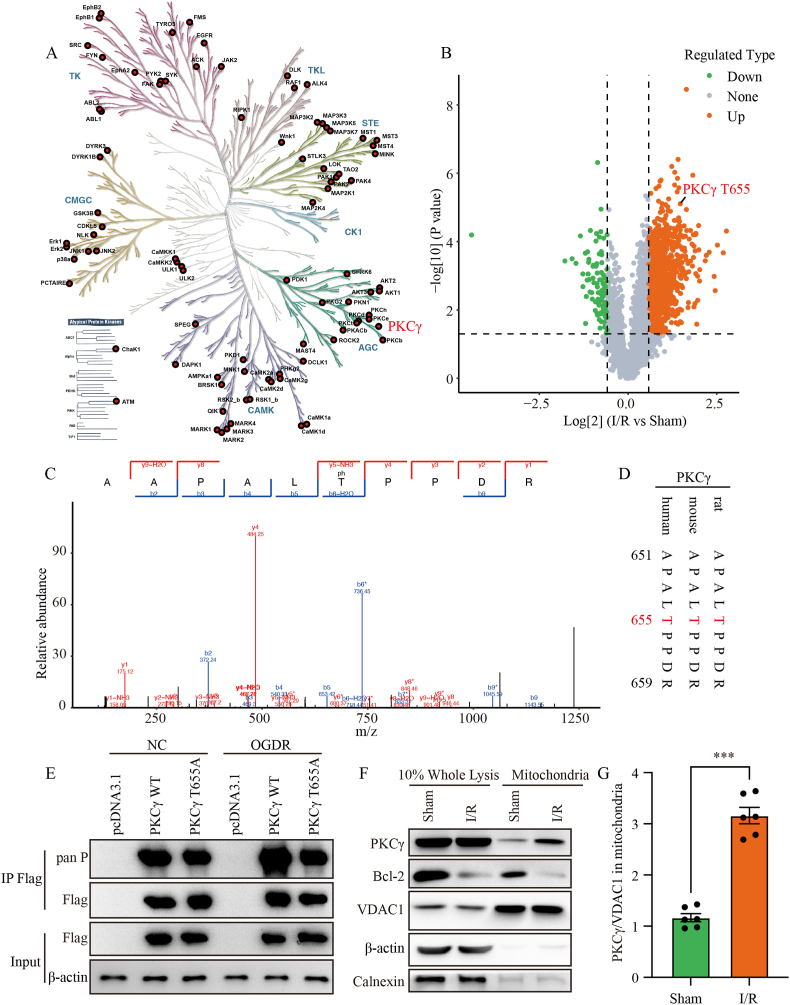
PKCγ phosphorylation is increased in the early stages of cerebral I/R. (A) Kinome map illustrating phosphopeptide kinases significantly altered after I/R. Different color branches represent distinct kinase families. (B) Volcano plot of differentially expressed phosphorylated mitochondrial proteins identified by mass spectrometry in mouse brain tissue following I/R. Orange dots represent significantly upregulated proteins, and green dots indicate significantly downregulated proteins. Notably, phosphorylation at the PKCγ T655 site is markedly increased. (C) Mass spectrometry spectrum of the PKCγ T655 phosphorylation site. The x-axis shows the mass-to-charge ratio (*m*/*z*), and the y-axis indicates peptide signal intensity (peak height reflects peptide abundance). (D) Sequence alignment of the amino acids surrounding T655 (highlighted in red) of PKCγ (positions 651–659) across different species, showing high conservation. (E) PKCγ was overexpressed in N2a cells (pcDNA3.1, PKCγ WT-Flag and PKCγ T655A-Flag). Following OGDR treatment, PKCγ was immunoprecipitated using anti-Flag beads, and its global phosphorylation was examined by Western blot. “IP” refers to the immunoprecipitated sample, and “Input” refers to the total cell lysate. (F) Western blot analysis of PKCγ protein expression in whole brain lysates (10% Whole Lysis) and isolated mitochondrial fractions from mice subjected to I/R. Bcl-2 was used as an anti-apoptotic protein. VDAC1 served as a mitochondrial marker, β-actin as a loading control, and Calnexin as an endoplasmic reticulum marker. n = 6. (G) Quantification of mitochondrial PKCγ expression in (F), normalized to VDAC1. OGDR, oxygen–glucose deprivation for 6 h followed by reoxygenation for 24 h (6 h/24 h); I/R, ischemia for 1 h followed by reperfusion for 24 h (1 h/24 h). ∗∗∗: p < 0.001.

To examine early signaling events during I/R, we focused on mitochondrial protein phosphorylation. Mitochondrial protein phosphorylation plays a crucial role in the pathological process of I/R. To investigate this, we utilized a transient MCAO mouse model to induce cerebral I/R and then performed phosphoproteomic analysis on isolated mitochondria. We identified multiple kinases whose phosphorylation levels were significantly altered following I/R (Fig. 1A). Notably, after label-free quantitation, PKCγ emerged as a key kinase with a significant increase in phosphorylation, indicating its potential activation. Specifically, phosphorylation at the PKCγ T655 site was markedly elevated (Fig. 1B and C). Sequence alignment further revealed that T655 is highly conserved across species (Fig. 1D). We next examined whether its phosphorylation status changes following OGDR *in vitro*. In N2a cells overexpressing PKCγ, we immunoprecipitated PKCγ after OGDR treatment and observed a pronounced increase in its overall phosphorylation (Fig. 1E). To further determine the role of the T655 site, we generated a T655A dephosphorylation mutant of PKCγ. Following OGDR treatment, immunoprecipitation of PKCγ-T655A combined with pan-phosphorylation detection showed that, in contrast to PKCγ WT, the OGDR-induced increase in overall phosphorylation was markedly abolished, indicating that mutation at the T655 site significantly attenuates the OGDR-induced phosphorylation signal of PKCγ. To further explore the subcellular localization of PKCγ after I/R, we isolated mitochondria from mouse brains and compared PKCγ protein levels between whole brain lysates and mitochondrial fractions. Western blot analysis showed following I/R, mitochondrial PKCγ expression increased significantly, accounting for approximately 5% of total PKCγ, suggesting its translocation to mitochondria and a potential role in the regulation of mitochondrial function (Fig. 1F and G).

### 3.2 Knockdown of PKCγ alleviates apoptosis following OGDR

**Fig. 2:**
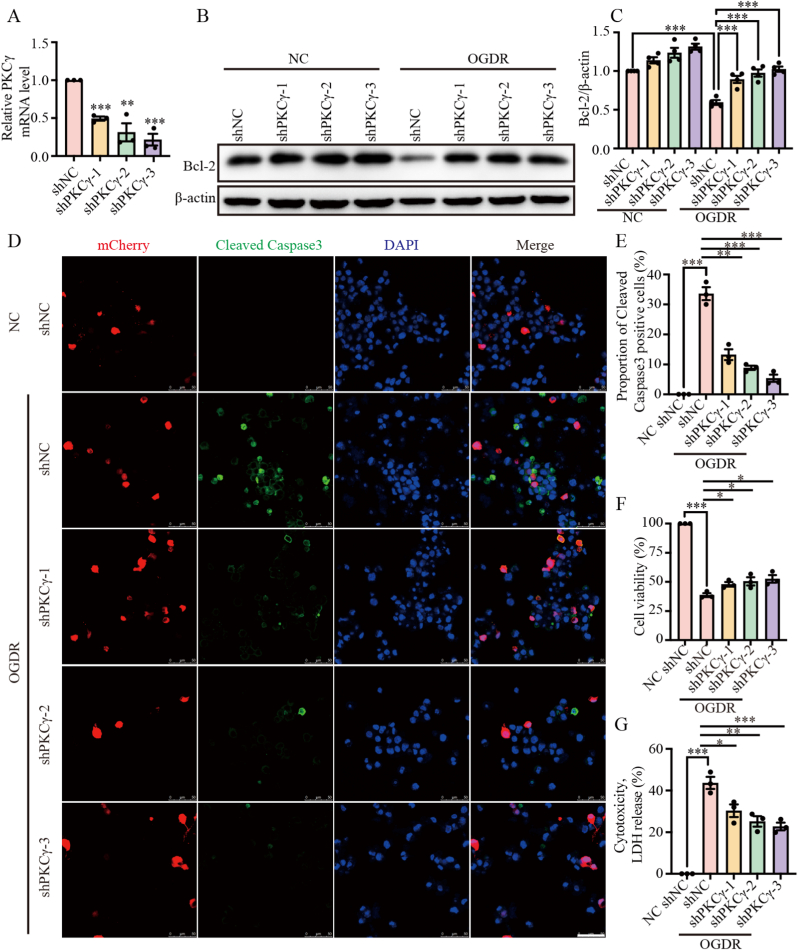
Knockdown of PKCγ alleviates apoptosis following OGDR. (A) qPCR analysis of PKCγ knockdown efficiency in N2a cells. Data are presented as mean ± SEM. (B) Western blot analysis of Bcl-2 expression in N2a cells with shRNA-mediated PKCγ knockdown following OGDR treatment. β-actin was used as a loading control. (C) Quantification of Bcl-2 expression normalized to β-actin. (D) Representative immunofluorescence images of Cleaved Caspase-3 in N2a cells following PKCγ knockdown after OGDR treatment. Red (mCherry) indicates cells transduced with shRNA, green marks Cleaved Caspase-3, and blue (DAPI) labels nuclei. Scale bar = 50 μm. (E) Quantification of (D), showing the percentage of Cleaved Caspase-3-positive cells. (F) CCK-8 assay measuring cell viability in PKCγ-knockdown N2a cells following OGDR. (G) LDH release assay assessing cell death in PKCγ-knockdown N2a cells after OGDR. OGDR, oxygen–glucose deprivation for 6 h followed by reoxygenation for 24 h (6 h/24 h). ∗: p < 0.05, ∗∗: p < 0.01, ∗∗∗: p < 0.001.

Cerebral I/R injury is frequently accompanied by cell apoptosis. To investigate the role of PKCγ in this process, we constructed three lentiviral plasmids targeting PKCγ and transduced N2a cells to knock down PKCγ expression. The knockdown efficiency was confirmed by qPCR (Fig. 2A). After OGDR treatment, Western blot analysis revealed that the anti-apoptotic protein Bcl-2 was significantly upregulated in PKCγ-knockdown cells (Fig. 2B and C). Additionally, immunofluorescence staining showed that silencing PKCγ decreased the proportion of Cleaved Caspase-3-positive cells under OGDR conditions (Fig. 2D and E). Consistent with these observations, both the CCK-8 assay (Fig. 2F) and the LDH release assay (Fig. 2G) demonstrated that PKCγ knockdown alleviated OGDR-induced cell death. Collectively, these findings suggest that inhibiting PKCγ confers a protective effect against OGDR-induced apoptosis.

### 3.3 PKCγ knockdown protects mitochondria and alleviates oxidative stress injury during OGDR

**Fig. 3:**
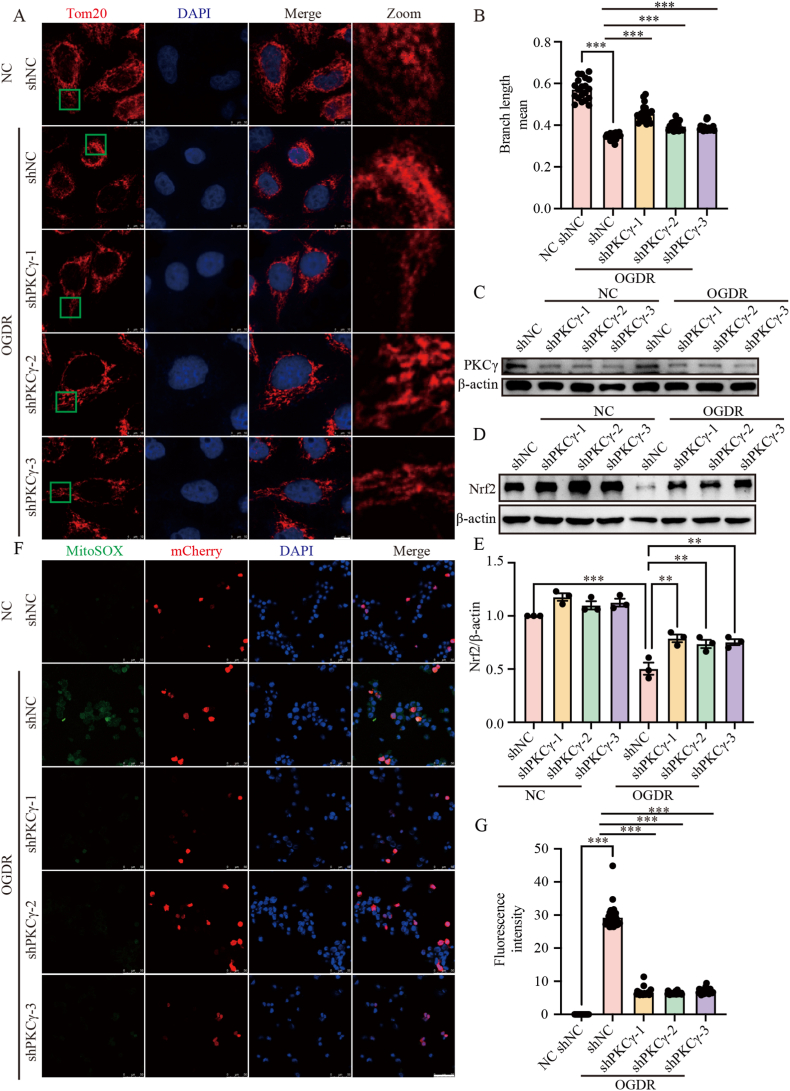
PKCγ knockdown protects mitochondria and alleviates oxidative stress injury during OGDR. (A) HeLa cells were transduced with shRNA against PKCγ, then subjected to OGDR. Mitochondrial morphology was assessed by immunofluorescence. Tom20 (red) labels mitochondria, and DAPI (blue) labels nuclei. Scale bar = 10 μm. The green box highlights a region shown in higher magnification (Zoom). (B) Quantification of mitochondrial branch length from (A). Data are presented as mean ± SEM. (C) HeLa cells were transfected with PKCγ shRNA, and the knockdown efficiency was assessed by Western blot analysis of PKCγ protein levels, with β-actin used as a loading control. (D)Western blot analysis of Nrf2 expression in N2a cells with shRNA-mediated PKCγ knockdown following OGDR. β-actin was used as a loading control. (E) Densitometric quantification of Nrf2 relative to β-actin from (D). (F) Representative immunofluorescence images of MitoSOX staining in N2a cells transduced with shRNA against PKCγ after OGDR. MitoSOX (green) indicates mitochondrial ROS, mCherry (red) labels shRNA-expressing cells, and DAPI (blue) stains nuclei. Scale bar = 50 μm. (G) Quantification of MitoSOX fluorescence intensity from (F). OGDR, oxygen–glucose deprivation for 6 h followed by reoxygenation for 24 h (6 h/24 h). ∗∗: p < 0.01, ∗∗∗:p < 0.001.

Mitochondrial dysfunction is central to the pathogenesis of I/R injury. As the primary organelle for energy metabolism, preserving mitochondrial integrity is crucial for normal cellular function. To investigate whether PKCγ knockdown affects mitochondrial morphology following OGDR, we transduced HeLa cells with shRNA targeting PKCγ and performed immunofluorescence staining. The results showed that mitochondrial fragmentation was significantly reduced in PKCγ-knockdown cells compared to controls (Fig. 3A–C), indicating that inhibiting PKCγ preserves mitochondrial structure under OGDR conditions. Excessive ROS production is another hallmark of I/R injury, often overwhelming cellular antioxidant defenses and exacerbating mitochondrial damage. Since PKCγ knockdown appeared to protect mitochondrial structure, we next examined whether PKCγ knockdown also mitigates oxidative stress. Nrf2 is a key transcription factor that regulates the antioxidant stress response, and its activation helps maintain mitochondrial function and reduce cell apoptosis [3]. In N2a cells, Western blot analysis revealed that PKCγ knockdown led to a significant increase in Nrf2 expression following OGDR (Fig. 3D and E). Consistent with these data, immunofluorescence detection of mitochondrial ROS using MitoSOX demonstrated a marked decrease in ROS levels in PKCγ-knockdown cells (Fig. 3F and G). Taken together, these findings suggest that inhibiting PKCγ enhances antioxidant defenses and helps maintain mitochondrial integrity after I/R, although the precise mechanisms remain to be elucidated.

### **3.4** *PKCγ T655 phosphorylation promotes apoptosis following OGDR*

**Fig. 4:**
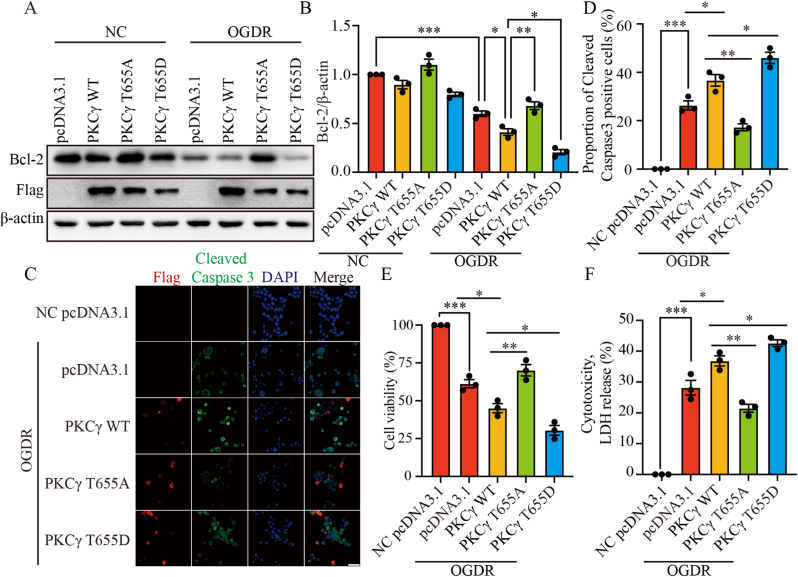
PKCγ T655 phosphorylation promotes apoptosis following OGDR. (A) N2a cells were transfected with pcDNA3.1, PKCγ WT-Flag, PKCγ T655A-Flag, or PKCγ T655D-Flag. After OGDR treatment, Western blot analysis was performed to assess the impact of PKCγ phosphorylation on Bcl-2 expression. β-actin was used as the loading control. (B) Quantification of (A), showing the ratio of Bcl-2 to β-actin. (C) Representative immunofluorescence images of Cleaved Caspase-3 in N2a cells transfected with the indicated constructs after OGDR. Flag (red), Cleaved Caspase-3 (green), and DAPI (blue). Scale bar = 50 μm. (D) Quantification of (C), showing the percentage of Cleaved Caspase-3-positive cells. (E) CCK-8 assay measuring cell viability in N2a cells transfected with pcDNA3.1, PKCγ WT, T655A, or T655D after OGDR. (F) LDH release assay assessing cell death under the same conditions. OGDR, oxygen–glucose deprivation for 6 h followed by reoxygenation for 24 h (6 h/24 h). ∗: p < 0.05, ∗∗: p < 0.01, ∗∗∗: p < 0.001.

To further examine the functional role of PKCγ phosphorylation during I/R injury, we focused on T655, which was identified as a key phosphorylation site by phosphoproteomics. We generated dephospho-mimetic (T655A) and phospho-mimetic (T655D) PKCγ mutants and compared their effects in N2a cells under OGDR conditions. N2a cells were transfected with pcDNA3.1, PKCγ WT, T655A, or T655D constructs, followed by OGDR treatment. Western blot analysis showed that overexpression of PKCγ WT or T655D downregulated Bcl-2 expression and aggravated OGDR-induced apoptotic damage, whereas overexpression of T655A produced a contrasting outcome (Fig. 4A and B). Immunofluorescence staining further revealed that PKCγ WT and T655D increased the proportion of Cleaved Caspase-3–positive cells, while T655A significantly reduced the proportion of Cleaved Caspase-3–positive cells (Fig. 4C and D). Consistently, CCK-8 cell viability assays (Fig. 4E) and LDH release assays (Fig. 4F) demonstrated that PKCγ WT and T655D promoted OGDR-induced cell death, whereas T655A resulted in an inverse effect. These findings suggest that phosphorylation of the PKCγ T655 site plays an important role in promoting OGDR-induced apoptosis.

### 3.5 PKCγ T655 phosphorylation impairs mitochondria and promotes oxidative stress injury during OGDR

**Fig. 5:**
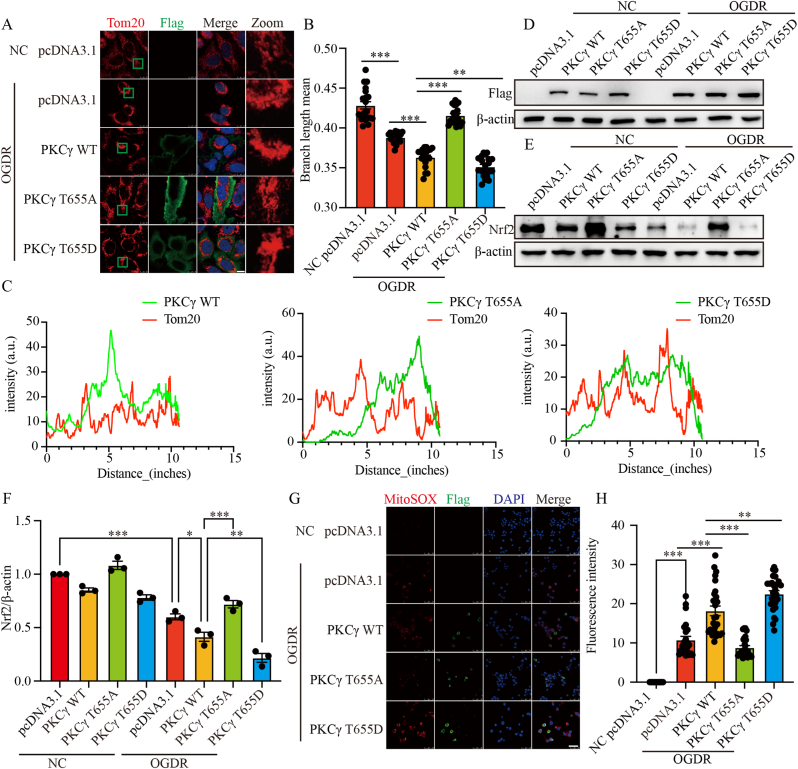
PKCγ T655 phosphorylation impairs mitochondria and promotes oxidative stress injury during OGDR. (A) HeLa cells were transfected with pcDNA3.1, PKCγ WT-Flag, PKCγ T655A-Flag, or PKCγ T655D-Flag. After OGDR treatment, mitochondrial morphology was assessed by immunofluorescence. Tom20 (red) labels mitochondria, Flag (green) labels the overexpressed constructs, and DAPI (blue) stains nuclei. Scale bar = 10 μm. The green box highlights a region shown at higher magnification (Zoom). (B) Quantification of mitochondrial branch length from (A). (C) Colocalization analysis of PKCγ (Flag) and mitochondria (Tom20) in (A). (D) HeLa cells were transfected with pcDNA3.1, PKCγ WT, T655A, or T655D constructs, and protein expression levels were analyzed by Western blot, with β-actin used as a loading control. (E) Western blot analysis of Nrf2 expression in N2a cells overexpressing pcDNA3.1, PKCγ WT, T655A, or T655D following OGDR. β-actin was used as the loading control. (F) Quantification of Nrf2 protein expression from (E). (G) Representative immunofluorescence images of MitoSOX staining in N2a cells transfected with the indicated constructs following OGDR. MitoSOX (red) indicates mitochondrial ROS, Flag (green) labels the overexpressed constructs, and DAPI (blue) stains nuclei. Scale bar = 50 μm. (H) Quantification of MitoSOX fluorescence intensity from (G). OGDR, oxygen–glucose deprivation for 6 h followed by reoxygenation for 24 h (6 h/24 h). ∗: p < 0.05, ∗∗: p < 0.01, ∗∗∗: p < 0.001.

Given these effects on apoptosis, we next investigated mitochondrial and oxidative stress responses. We examined how PKCγ T655 mutations affect mitochondrial integrity. HeLa cells were transfected with pcDNA3.1, PKCγ WT, PKCγ T655A, or PKCγ T655D constructs, followed by OGDR treatment. Immunofluorescence analysis revealed that overexpression of PKCγ WT or T655D significantly promoted mitochondrial fragmentation, whereas T655A led to a reversed response (Fig. 5A and B). These findings suggest that phosphorylation at PKCγ T655 promotes OGDR-induced mitochondrial damage. Based on these alterations in mitochondrial integrity, we next analyzed the subcellular localization of PKCγ. Immunofluorescence colocalization of PKCγ (Flag) with the mitochondrial marker Tom20 demonstrated that PKCγ WT exhibited clear mitochondrial colocalization, which was further enhanced in the T655D mutant, whereas the T655A mutant showed markedly reduced colocalization with mitochondria (Fig. 5C and D), suggesting that phosphorylation at T655 promotes PKCγ translocation to mitochondria. We next explored the relationship between PKCγ T655 phosphorylation and oxidative stress. In N2a cells, Western blot analysis showed that overexpression of PKCγ WT or T655D further suppressed Nrf2 expression following OGDR treatment, whereas T655A elicited a contrary effect (Fig. 5E and F). Consistently, mitochondrial ROS levels detected by immunofluorescence using MitoSOX indicated increased ROS in cells overexpressing PKCγ WT or T655D, whereas overexpression of T655A showed a diametrically opposed result (Fig. 5G and H). Taken together, these results demonstrate that phosphorylation at the PKCγ T655 site exacerbates oxidative stress and mitochondrial damage. Therefore, targeting the phosphorylation status of PKCγ T655 may represent a potential target for modulating cerebral I/R injury.

### **3.6** *PKCγ regulates oxidative stress and apoptosis by modulating Nrf2 expression and nuclear translocation*

**Fig. 6:**
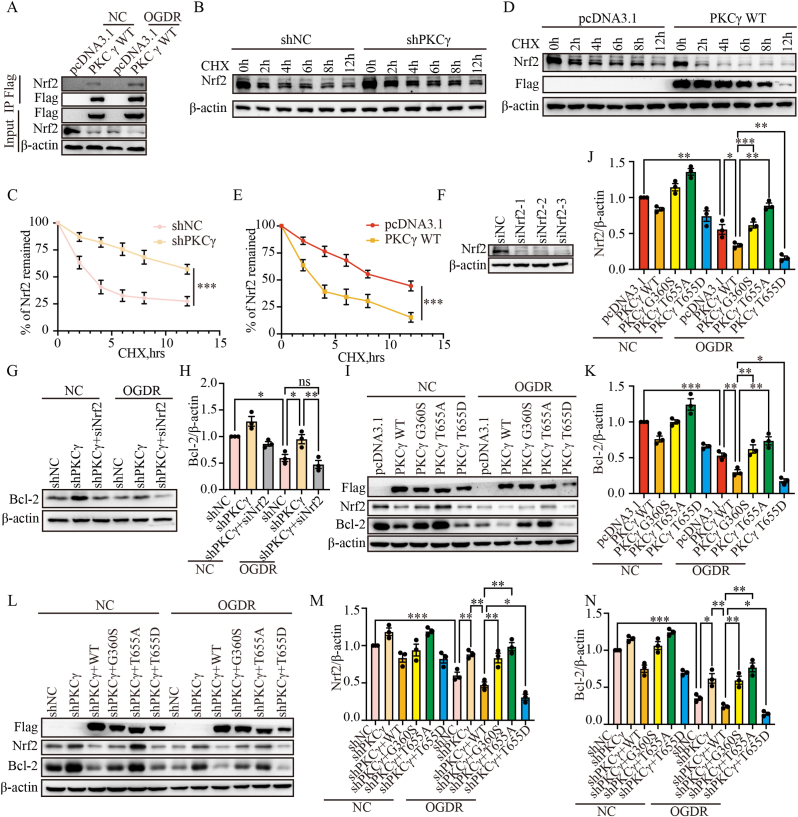
PKCγ regulates Nrf2 expression and protein stability. (A) PKCγ was overexpressed in N2a cells (pcDNA3.1 and PKCγ WT-Flag). Following OGDR treatment, PKCγ was immunoprecipitated using anti-Flag beads, and endogenous Nrf2 expression was examined by Western blot. “IP” refers to the immunoprecipitated sample, and “Input” refers to the total cell lysate. (B) PKCγ was knocked down in N2a cells using shRNA (shPKCγ-1). Following 100 μg/mL CHX treatment for the indicated time points (0, 2, 4, 6, 8, and 12 h), Nrf2 expression was examined by Western blot. (C) Quantification of relative Nrf2 levels under the different treatment conditions shown in (B). (D) PKCγ was overexpressed in N2a cells (pcDNA3.1 and PKCγ WT-Flag). Following CHX treatment for the indicated time points, Nrf2 expression was examined by Western blot. (E) Quantification of relative Nrf2 levels under the different treatment conditions shown in (D). (F) Western blot analysis of Nrf2 knockdown in N2a cells, with β-actin as a loading control. (G) Expression of Bcl-2 in N2a cells after OGDR following knockdown of PKCγ (shPKCγ-1) and Nrf2 (siNrf2-3), with β-actin as a loading control. (H) Quantification of the Bcl-2/β-actin ratio from (G). (I) Western blot analysis of Nrf2 and Bcl-2 protein levels in N2a cells overexpressing pcDNA3.1, PKCγ WT, G360S, T655A, or T655D following OGDR. β-actin was used as the loading control. (J) Quantification of Nrf2 protein levels relative to β-actin from (I). (K) Quantification of Bcl-2 protein levels relative to β-actin from (I). (L) Western blot analysis of Nrf2 and Bcl-2 expression in PKCγ knockdown N2a cells (via shPKCγ-1) overexpressing PKCγ WT, G360S, T655A, or T655D following OGDR. β-actin was used as the loading control. (M) Quantification of Nrf2 protein levels relative to β-actin from (L). (N) Quantification of Bcl-2 protein levels relative to β-actin from (L). OGDR, oxygen–glucose deprivation for 6 h followed by reoxygenation for 24 h (6 h/24 h). ns, not significant, ∗: p < 0.05, ∗∗: p < 0.01, ∗∗∗: p < 0.001.

To investigate how PKCγ regulates Nrf2-mediated oxidative stress and apoptosis following I/R, we performed co-immunoprecipitation assays in N2a cells overexpressing PKCγ after OGDR treatment and observed an increased association between PKCγ and Nrf2 (Fig. 6A). We then examined the half-life of Nrf2 using protein synthesis inhibitor CHX chase assays and found that knockdown of PKCγ delayed Nrf2 protein degradation (Fig. 6B and C), whereas PKCγ overexpression accelerated Nrf2 degradation (Fig. 6D and E). These findings suggest that PKCγ negatively regulates Nrf2 protein stability.

To determine whether the protective effects induced by PKCγ knockdown are functionally associated with Nrf2 signaling, we further performed Nrf2 silencing in the context of PKCγ knockdown. Under OGDR conditions, N2a cells were co-transfected with shPKCγ and siNrf2. Western blot analysis showed that shPKCγ markedly increased the expression of the anti-apoptotic protein Bcl-2, whereas this upregulation was substantially attenuated when Nrf2 was suppressed (Fig. 6F–H). These results indicate that the anti-apoptotic effect conferred by PKCγ knockdown is, at least partially dependent on Nrf2, supporting a functional regulatory axis between PKCγ and Nrf2 in the context of ischemia–reperfusion injury.

To further explore the role of PKCγ phosphorylation, we focused on the T655 site identified by phosphoproteomic analysis as being upregulated following I/R injury. We also generated a kinase-dead mutant (G360S) as a control [30]. Next, we further investigated the effects of PKCγ mutants on Nrf2 protein levels and the anti-apoptotic protein Bcl-2. After overexpressing the mutants in N2a cells, Western blot analysis revealed that both WT and the phospho-mimetic mutant T655D significantly suppressed the protein levels of Nrf2 and Bcl-2, whereas the kinase-dead mutant G360S and the dephospho-mimetic mutant T655A induced an opposing response (Fig. 6I–K). Rescue experiments further demonstrated that re-expression of PKCγ WT or T655D, but not T655A or G360S, reversed the upregulation of Nrf2 and Bcl-2 induced by PKCγ knockdown (Fig. 6L–N). These findings support a functional role of T655 phosphorylation in modulating PKCγ activity and its downstream effects on Nrf2 signaling.

**Fig. 7:**
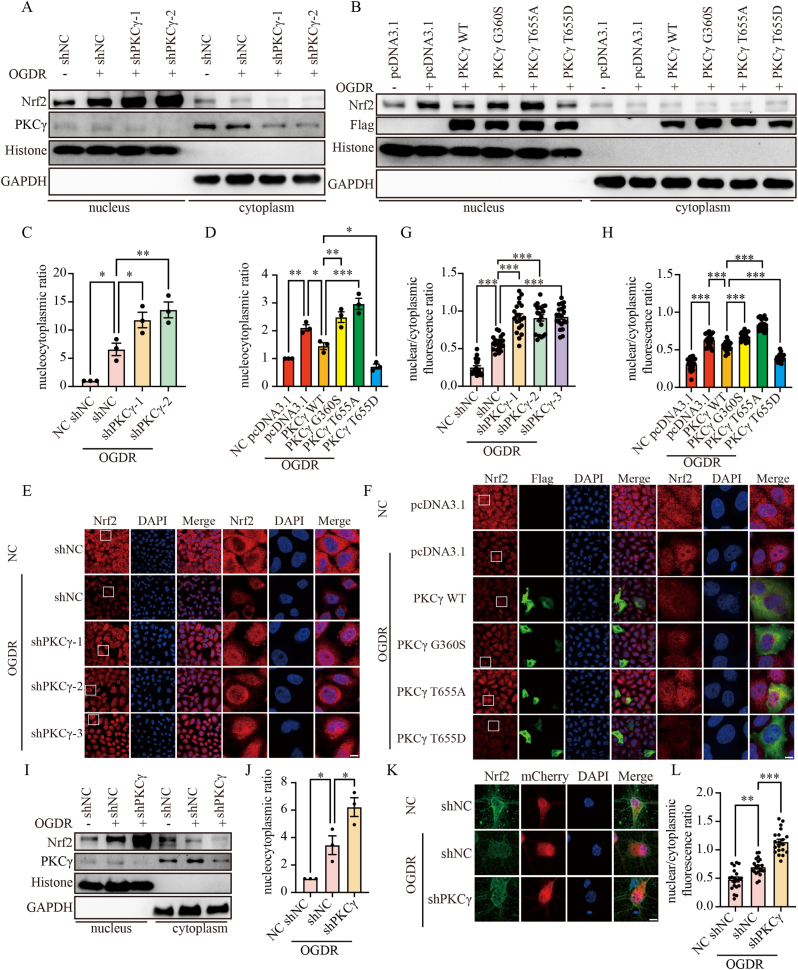
PKCγ regulates Nrf2 nuclear translocation. (A) HeLa cells were transduced with shRNA targeting PKCγ and subjected to OGDR. Nuclear and cytoplasmic fractions were isolated, and Nrf2 expression in each fraction was detected by Western blot. Histone and GAPDH were used as nuclear and cytoplasmic markers, respectively. (B) HeLa cells were transfected with PKCγ WT, G360S, T655A, or T655D plasmids and subjected to OGDR. Nuclear and cytoplasmic fractions were analyzed by Western blot for Nrf2 expression, with Histone and GAPDH as nuclear and cytoplasmic markers. (C) Quantification of the nuclear-to-cytoplasmic ratio of Nrf2 from (A). (D) Quantification of the nuclear-to-cytoplasmic ratio of Nrf2 from (B). (E) HeLa cells were transduced with shRNA against PKCγ and subjected to OGDR. Nrf2 subcellular localization was examined by immunofluorescence. Cy3 (red) labels Nrf2, and DAPI (blue) labels nuclei. Scale bar = 10 μm. The white box highlights a region shown at higher magnification. (F) HeLa cells were transfected with pcDNA3.1, PKCγ WT, G360S, T655A, or T655D plasmids and subjected to OGDR. Nrf2 subcellular localization was examined by immunofluorescence. Cy3 (red) labels Nrf2, Flag (green) labels the overexpressed constructs, and DAPI (blue) labels nuclei. Scale bar = 10 μm. The white box highlights a region shown at higher magnification. (G) Quantification of the nuclear-to-cytoplasmic ratio of Nrf2 fluorescence intensity from (E), n = 20. (H) Quantification of the nuclear-to-cytoplasmic ratio of Nrf2 fluorescence intensity from (F), n = 20. (I) Primary neurons were infected with AAV to knock down PKCγ and subjected to OGDR. Nuclear and cytoplasmic fractions were analyzed by Western blot for Nrf2 expression, with Histone and GAPDH as nuclear and cytoplasmic markers. (J) Quantification of the nuclear-to-cytoplasmic ratio of Nrf2 from (I). (K) AAV-infected neurons with PKCγ knockdown were subjected to OGDR and analyzed by immunofluorescence. Nrf2 is labeled in green, AAV-infected neurons in red, and DAPI (blue) labels nuclei. Scale bar = 25 μm. (L) Quantification of Nrf2 nuclear localization from (K), n = 20. OGDR, oxygen–glucose deprivation for 6 h followed by reoxygenation for 24 h (HeLa cells, 6 h/24 h) or 2 h followed by reoxygenation for 24 h (primary neurons, 2 h/24 h). ∗: p < 0.05, ∗∗: p < 0.01, ∗∗∗: p < 0.001.

Given that nuclear translocation of Nrf2 is a critical hallmark of its activation [31], we further investigated whether PKCγ participates in regulating the subcellular localization of Nrf2. In HeLa cells, PKCγ knockdown followed by OGDR treatment and nuclear/cytoplasmic fractionation with Western blot analysis revealed that PKCγ knockdown significantly increased Nrf2 accumulation in the nucleus (Fig. 7A and C). Upon transfection with PKCγ WT, G360S, T655A, or T655D, WT and T655D markedly suppressed Nrf2 nuclear translocation, whereas T655A and G360S failed to inhibit it (Fig. 7B and D), indicating that phosphorylation at the T655 site is critical for PKCγ activity and its downstream regulation of Nrf2 signaling. Immunofluorescence analysis further confirmed that Nrf2 underwent nuclear translocation after OGDR treatment in HeLa cells, and PKCγ knockdown significantly enhanced this effect (Fig. 7E and G), suggesting that inhibition of PKCγ facilitates Nrf2 accumulation in the nucleus, thereby enhancing its antioxidant function. Subsequently, HeLa cells were transfected to overexpress pcDNA3.1, PKCγ WT, G360S, T655A, or T655D, and the subcellular distribution of Nrf2 after OGDR treatment was examined. The results showed that PKCγ WT and T655D significantly suppressed Nrf2 nuclear translocation, whereas T655A and G360S mutants failed to inhibit it (Fig. 7F and H). Importantly, consistent results were observed in primary neurons. AAV-mediated knockdown of PKCγ significantly increased nuclear accumulation of Nrf2 under OGDR conditions, as shown by both nuclear/cytoplasmic fractionation and immunofluorescence analysis (Fig. 7I–L). These findings indicate that PKCγ regulates Nrf2 protein stability and nuclear translocation through phosphorylation at the T655 site, thereby influencing oxidative stress and apoptosis, providing mechanistic evidence for its key regulatory role in I/R injury.

### **3.7** *shPKCγ AAV attenuates mitochondrial damage and apoptosis after I/R*

**Fig. 8:**
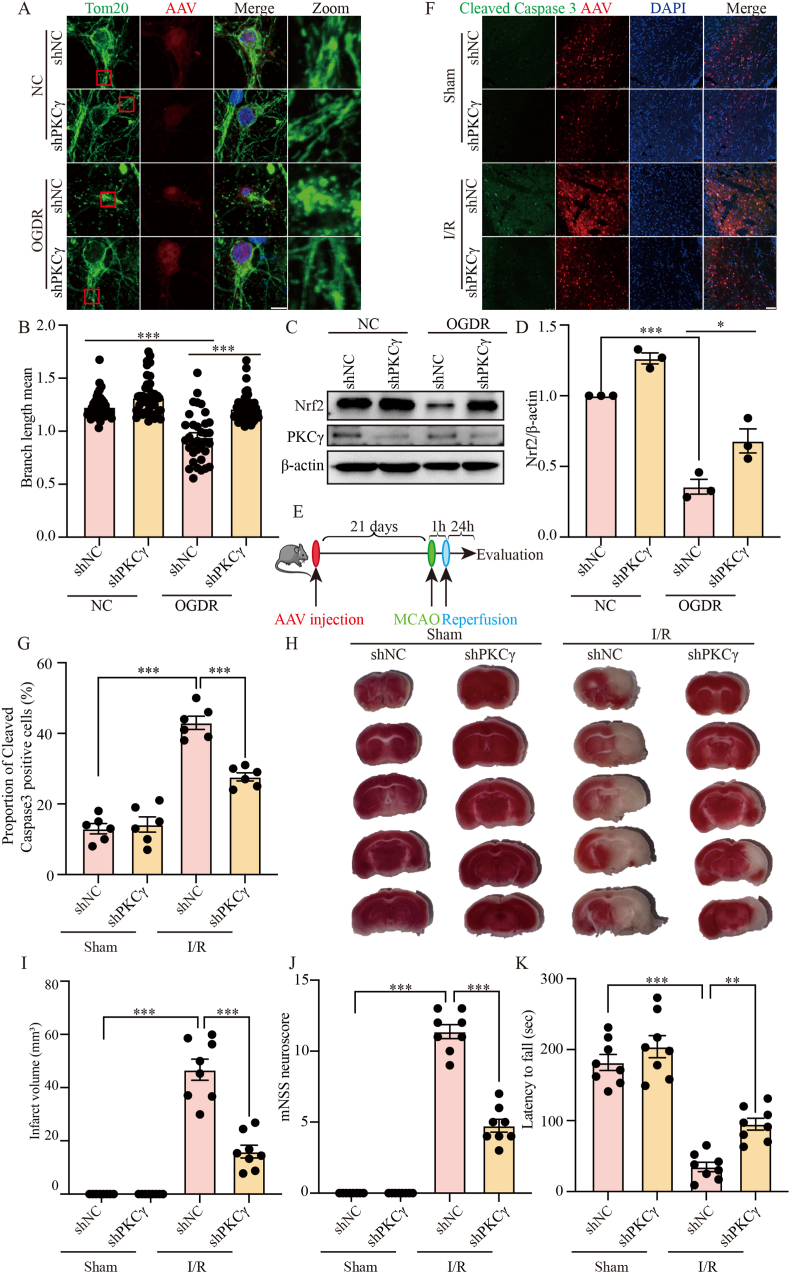
shPKCγ AAV knockdown attenuates mitochondrial damage and apoptosis after I/R. (A) Representative immunofluorescence images of primary mouse cortical neurons transduced with shNC AAV or shPKCγ AAV following OGDR. Tom20 (green) labels mitochondria, AAV-infected neurons are labeled in red, and DAPI (blue) labels nuclei. Scale bar = 25 μm. The red box highlights a magnified region (Zoom). (B) Quantification of mitochondrial branch length from (A). (C) Western blot analysis of Nrf2 expression in primary neurons transduced with shNC AAV or shPKCγ AAV after OGDR. β-actin serves as the loading control. (D) Densitometric quantification of Nrf2 relative to β-actin. (E) Schematic illustration of the experimental protocol for shPKCγ AAV injection in the MCAO mouse model. (F) Representative immunofluorescence images of Cleaved Caspase-3 (green) in brain tissue from mice injected with shNC AAV or shPKCγ AAV after I/R. Red labels AAV-infected neurons, and nuclei are stained with DAPI (blue). Scale bar = 50 μm. (G) Quantification of Cleaved Caspase-3-positive cells from (F), n = 6. (H) Representative TTC-stained brain sections from mice injected with shNC AAV or shPKCγ AAV following I/R. White regions indicate infarcted (ischemic) areas, and red regions represent viable brain tissue. (I) Quantification of infarct volume from (H). (J) Neurological deficits assessed using the mNSS at day 1 after MCAO in mice injected with shNC AAV or shPKCγ AAV (n = 8 per group). (K) Rotarod test evaluating motor coordination at day 1 post-MCAO. Latency to fall is shown (n = 8 per group). OGDR, oxygen–glucose deprivation for 2 h followed by reoxygenation for 24 h ((primary neurons, 2 h/24 h)); I/R, ischemia for 1 h followed by reperfusion for 24 h (1 h/24 h). ∗: p < 0.05, ∗∗: p < 0.01, ∗∗∗: p < 0.001.

To investigate whether this neuroprotective effect extends to an *in vivo* context, we transduced primary mouse cortical neurons with either control (shNC) or shPKCγ AAV. Under OGDR conditions, shPKCγ significantly reduced mitochondrial fragmentation (Fig. 8A and B) and upregulated Nrf2 expression (Fig. 8C and D), indicating improved resistance to oxidative stress. We next evaluated the *in vivo* effect of PKCγ knockdown by stereotactically injecting shPKCγ AAV into the bilateral S1FL region (X = ±2.0 mm, Y = −0.22 mm, Z = −1.8 mm) of the mouse cortex (Fig. 8E). After three weeks to allow for robust viral expression, mice underwent I/R. Immunofluorescence analysis of brain sections revealed that shPKCγ AAV significantly reduced the number of Cleaved Caspase-3-positive cells compared to shNC AAV (Fig. 8F and G). To further evaluate the therapeutic efficacy of PKCγ knockdown *in vivo*, we assessed key indicators of brain injury and neurological function. TTC staining showed that mice infected with shPKCγ AAV exhibited significantly reduced infarct volumes compared to the shNC AAV group (Fig. 8H and I), indicating alleviated brain injury after I/R. Consistently, behavioral assessments confirmed significant improvements in neurological outcomes: mNSS scores demonstrated significantly better neurological function (Fig. 8J), and rotarod tests indicated enhanced motor coordination in the shPKCγ AAV group (Fig. 8K). Taken together, these results suggest that AAV-mediated PKCγ knockdown not only mitigates mitochondrial damage and apoptosis but also reduces cerebral infarction and improves neurological and motor functions after I/R, supporting its translational potential as a therapeutic strategy for ischemic stroke.

## 4 Discussion

PKC represents a family of serine-threonine kinases that regulate a wide range of cellular processes. This family comprises 11 genes encoding structurally related phospholipid-dependent kinases with different regulatory and tissue distribution patterns. Based on their structure and sensitivity to Ca^2+^ and diacylglycerol (DAG), they are classified into conventional PKCs (α, βI, βII, and γ), novel PKCs (δ, ε, η, and θ), and atypical PKCs (aPKC: ζ and λ/ι) [32]. PKCλ and PKCι are orthologs of the same enzyme in mice and humans, respectively [33]. Conventional PKCs are activated by both DAG and Ca^2+^, novel PKCs are activated by DAG but not Ca^2+^, and atypical PKCs are insensitive to both DAG and Ca^2+^. Among conventional PKCs, α and β isoforms primarily function in non-neuronal tissues to regulate proliferation and apoptosis [[34], [35], [36], [37]]. PKCδ and PKCε are critical in oxidative stress and inflammatory responses [38]. The aPKC isoenzymes, identified as oncogenes, play key roles in cancer and insulin signaling [39,40]. In the context of cerebral I/R injury, different PKC isoforms exhibit divergent and sometimes opposing roles [41]. PKCδ is generally considered pro-apoptotic and detrimental; it mediates neuronal death by promoting mitochondrial cytochrome *c* release, activating caspase cascades, and disrupting the blood-brain barrier (BBB) integrity [42]. Inhibition of PKCδ has been shown to reduce infarct size and improve neurological outcomes in rodent stroke models [43]. Conversely, PKCε has been demonstrated to exert neuroprotective effects during cerebral ischemia. It preserves mitochondrial function by regulating mitochondrial permeability transition pores and enhances anti-apoptotic signaling pathways [44]. Moreover, PKCε activation attenuates neuroinflammation by inhibiting microglial activation and pro-inflammatory cytokine release [45]. Thus, PKCε serves as an important endogenous neuroprotective kinase in ischemic brain injury. The atypical PKCs, including PKCζ and PKCι, are less studied in stroke but are implicated in maintaining BBB integrity and neurovascular remodeling post-injury. PKCζ contributes to endothelial tight junction protein phosphorylation, promoting barrier function recovery after ischemic insult [46]. PKCι also participates in cytoskeletal reorganization necessary for angiogenesis and repair [47]. Among PKC isoforms, PKCγ is particularly noteworthy due to its near-exclusive neuronal expression, predominantly in the cerebellum, hippocampus, and cerebral cortex [48], and activation by Ca^2+^, DAG, and free fatty acids [[49], [50], [51]]. This neural specificity underpins its unique significance in cerebral I/R injury. In addition to its established roles in long-term potentiation and spatial memory [52], point mutations in the PKCγ gene have been linked to Parkinson's syndrome [53] and spinocerebellar ataxia [54].

Although previous studies have reported the translocation of PKCγ to cell membranes under cerebral ischemic conditions [[55], [56], [57]], the "membrane fractions" analyzed in those studies were often crude preparations obtained through subcellular fractionation methods such as differential centrifugation. These fractions contained a mixture of components, including mitochondria and endoplasmic reticulum, and were susceptible to contamination by synaptosomes and myelin, complicating precise subcellular localization of PKCγ. In contrast, our study adopted the method reported by Sims et al. [24], using Percoll density gradient centrifugation to isolate highly purified mitochondrial fractions, substantially reducing contamination. Unlike previous studies that primarily assessed PKCγ translocation using crude membrane fractions, our study combined highly purified mitochondrial fractionation, phosphoproteomics, and functional validation. These results, building on previous studies, reveal the specific role of PKCγ phosphorylation in mitochondrial localization, provide mechanistic insights, and support a direct causal link between PKCγ phosphorylation, oxidative stress, and neuronal injury. Functional experiments showed that PKCγ knockdown significantly alleviated neuronal apoptosis and mitochondrial dysfunction, supporting PKCγ inhibition or specific targeting of T655 phosphorylation as a potential neuroprotective strategy.

Beyond mitochondrial localization, PKCγ activation modulates oxidative stress by regulating ROS generation [58]. Once activated, PKCγ alters mitochondrial membrane permeability or disrupts mitochondrial Ca^2+^ homeostasis, exacerbating oxidative stress and impairing mitochondrial function [59]. Nrf2, a master regulator of antioxidant responses, translocates to the nucleus upon activation, binds small Maf (sMaf) proteins and the antioxidant response element (ARE) [60,61], and induces expression of antioxidant genes such as *HO-1* and *NQO1* [62,63]. Oxidative stress plays a central role in the initiation and progression of cerebral I/R injury. The Keap1–Nrf2 pathway is a core cellular antioxidant defense mechanism: under basal conditions, Keap1 promotes Nrf2 ubiquitination and proteasomal degradation, whereas oxidative stress induces conformational changes in Keap1 that inhibit Nrf2 degradation, stabilizing Nrf2 and enabling its nuclear translocation to activate antioxidant and cytoprotective genes, including *HO-1*, *NQO1*, and *GCLC* [3].

**Fig. S1:**
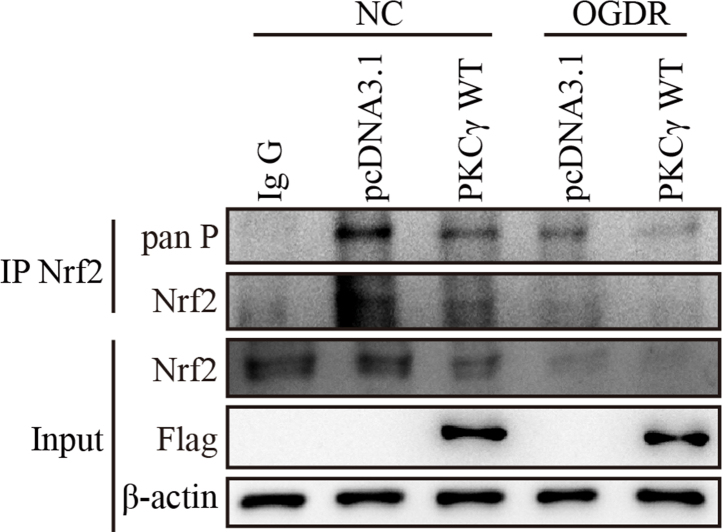
PKCγ does not induce phosphorylation of Nrf2 following OGDR treatment. In N2a cells, pcDNA3.1 or PKCγ WT-Flag was transfected. Following OGDR treatment, Nrf2 was immunoprecipitated using agarose beads, and its global phosphorylation level was assessed by Western blot. “IP” denotes the immunoprecipitated fraction, “Input” refers to total cell lysate, and IgG was used as a negative control.

Western blot and CHX chase assays indicated that PKCγ negatively regulates Nrf2 stability, whereas PKCγ knockdown prolongs Nrf2 half-life and enhances its protective effect. PKCγ knockdown also increased Bcl-2 expression, partially reversed by Nrf2 silencing, suggesting a functional PKCγ–Nrf2 axis. Nuclear/cytoplasmic fractionation and immunofluorescence analyses in HeLa cells and primary neurons demonstrated that PKCγ knockdown significantly promoted Nrf2 nuclear accumulation following OGDR treatment. Re-expression of PKCγ WT or the phospho-mimetic T655D mutant suppressed Nrf2 nuclear translocation, whereas dephospho-mimetic T655A and kinase-dead G360S mutants failed to do so, providing direct evidence that T655 phosphorylation modulates Nrf2 localization and antioxidant function, linking subcellular positioning to oxidative stress and apoptosis. These results delineate a clear mechanistic pathway, in which phosphorylation at the T655 site regulates PKCγ kinase activity, thereby influencing Nrf2 nuclear translocation, antioxidant gene expression, and mitochondrial ROS levels, ultimately determining neuronal survival. Notably, our immunoprecipitation analysis showed that PKCγ overexpression did not alter the global phosphorylation level of Nrf2 following OGDR treatment (Fig. S1), suggesting that PKCγ is unlikely to directly phosphorylate Nrf2. Instead, PKCγ may regulate Nrf2 activity through indirect mechanisms, such as modulation of its stability or upstream signaling pathways.

Further functional experiments highlighted the critical role of T655 phosphorylation for PKCγ kinase activity. The T655D mutant suppressed Nrf2 expression and nuclear translocation, increased mitochondrial ROS, and exacerbated neuronal apoptosis, whereas T655A produced opposite effects. Rescue experiments confirmed that only WT or T655D re-expression reversed Nrf2 and Bcl-2 upregulation caused by PKCγ knockdown, emphasizing T655 phosphorylation as a key mechanism through which PKCγ regulates Nrf2 activity, oxidative stress, mitochondrial homeostasis, and neuronal survival.

Notably, mitochondrial damage involves not only ROS generation and cytochrome *c* release but also mitophagy, which is critical for maintaining mitochondrial quality. The timing and extent of mitophagic responses during reperfusion remain to be clarified and warrant further investigation in future studies. Moreover, to ensure comparability between the *in vitro* experiments and the *in vivo* I/R model, the duration of OGDR treatment *in vitro* was optimized according to the injury characteristics observed *in vivo*, and validated using nuclear/cytoplasmic fractionation combined with immunofluorescence analyses. It should be noted, however, that the OGDR and I/R time paradigms differ across experimental systems in this study, including HeLa cells, primary neurons, and the *in vivo* model. This variation primarily reflects the distinct sensitivities of different cell types and tissues to hypoxic/ischemic stress, rather than representing directly comparable temporal scales. Importantly, despite these differences, the key observations—namely PKCγ activation, mitochondrial dysfunction, and suppression of Nrf2 signaling—show consistent directional changes across models. Therefore, cross-system comparisons in this study are based on the consistency of mechanistic trends rather than strict temporal equivalence. Nevertheless, we acknowledge that such temporal heterogeneity represents a limitation for precise mechanistic extrapolation between *in vitro* and *in vivo* systems. Future studies incorporating time-course alignment or dynamic monitoring approaches will be required to further refine the temporal relationship of PKCγ signaling across models. The concordance of these results across HeLa cells, primary neurons, and mouse cortical neurons strengthens the robustness of the proposed PKCγ–Nrf2 pathway in mediating oxidative stress and neuronal apoptosis. Importantly, *in vivo* experiments using AAV-mediated PKCγ knockdown further confirmed that PKCγ depletion reduces infarct size, decreases neuronal apoptosis, and improves neurological outcomes, directly supporting the translational relevance of our mechanistic findings from *in vitro* systems. Collectively, these results indicate that PKCγ signaling consistently regulates neuronal injury under ischemic stress and reveal a direct causal link from molecular modifications to cellular and tissue-level outcomes. Targeting PKCγ, particularly its phosphorylation at the T655 site, may represent a potential therapeutic strategy for cerebral ischemic injury.

Studying downstream substrates of PKCγ helps elucidate its neurobiological mechanisms. Under basal conditions, PKCγ interacts with various cellular scaffolding proteins to regulate gap junction communication via its exposed C1B domain, which normally binds DAG [64]. However, exposure to oxidative agents such as hydrogen peroxide oxidizes this domain, leading to disulfide bond formation and structural alterations [65]. C1B mutations may disrupt endogenous PKCγ binding with other relevant proteins, leading to inactivation of endogenous PKCγ and subsequent alterations in cellular signaling pathways, such as responses to gap junction-coupled stimuli. Apoptotic signals related to disrupted gap junctions indicate that malfunctioning endogenous PKCγ impedes the dynamic disassembly of gap junction plaques before and after oxidative stress, thereby triggering cell apoptosis. Thus, different phosphorylation sites direct diverse cellular outcomes for PKCγ. Additionally, PKCγ interacts with the highly conserved heat-shock protein 90α (Hsp90α) [66]. Newly synthesized PKCγ is unstable and requires Hsp90α, along with the co-chaperone Cdc37, to stabilize and translocate to the plasma membrane. Upon subsequent activation by second messengers, PKCγ dissociates from the Hsp90α chaperone complex and phosphorylates Hsp90α at serine residues (Thr 115, Thr 425, Thr 603), a process that promotes cell migration [48,67,68]. Furthermore, PKCγ phosphorylates connexin-43 (Cx43), a major gap junction protein widely expressed in neurons and astrocytes. Phosphorylation of Cx43 by PKCγ significantly alters intercellular communication and has been associated with neurotoxicity under oxidative stress conditions [69,70]. Additionally, Zhang et al. reported that other phosphorylation sites on PKCγ (such as T514 and T674) are associated with the pathogenesis of schizophrenia [71]. Our study highlights T655 as a key regulatory phosphorylation site in cerebral I/R injury, although it may not be the only critical site, as other yet-unidentified residues could also influence neuronal damage. Future studies should investigate additional PKCγ phosphorylation sites and their temporal regulation during reperfusion to fully delineate the upstream and downstream networks. Collectively, our data establish T655 as a central regulatory node and provide a foundation for precise therapeutic strategies targeting PKCγ phosphorylation to mitigate ischemic brain injury.

## 5 Conclusion

In conclusion, our study identifies phosphorylation at PKCγ T655 as a key regulator of its kinase activity, which suppresses Nrf2 signaling, increases mitochondrial oxidative stress, and promotes neuronal apoptosis during cerebral I/R injury. Both *in vitro* and *in vivo* models confirm that inhibition of PKCγ or prevention of T655 phosphorylation confers neuroprotection, reduces infarct size, and improves functional outcomes. These findings provide novel mechanistic insights and suggest that targeting PKCγ T655 phosphorylation may represent a promising therapeutic strategy for ischemic stroke.

## Availability of data and material

The original contributions presented in the study are included within the article, further inquiries can be directed to the corresponding authors.

## Ethical approval

All animal care and experimental procedures were reviewed and approved by the Ethics Committee of Experimental Animals, Xiangya Second Hospital, Central South University (Approval No. 20240673).

## Funding

This work was supported by grants from the Major Program of the National Natural Science Foundation (22494704 to J.T.), 10.13039/501100012166National Key Research and Development Program of China (no. 2021YFA0805200 to J.T.), the 10.13039/501100001809National Natural Science Foundation of China (8197052445 to L.Z) and the 10.13039/501100004735Hunan Provincial Natural Science Foundation (2025JJ50618 to L.Z).

## CRediT authorship contribution statement

**Chenchen Li:** Conceptualization, Data curation, Formal analysis, Investigation, Methodology, Software, Writing – original draft, Writing – review & editing. **Jinlun Chen:** Data curation, Methodology, Writing – review & editing. **Xiangbin Ouyang:** Data curation, Investigation, Writing – review & editing. **Ruijia Duan:** Data curation, Methodology, Writing – review & editing. **Yijin Kuang:** Data curation, Methodology, Writing – review & editing. **Yaohui He:** Conceptualization, Data curation, Funding acquisition, Methodology, Writing – review & editing. **Jieqiong Tan:** Conceptualization, Data curation, Funding acquisition, Methodology, Writing – review & editing. **Liuwang Zeng:** Conceptualization, Data curation, Funding acquisition, Methodology, Writing – review & editing.

## Declaration of competing interest

The authors have declared that no conflict of interest exists.

## Data Availability

Data will be made available on request.
